# Strengthening antimicrobial stewardship through an international multicentre improvement programme of hospitals serving deprived populations

**DOI:** 10.1093/jacamr/dlag241

**Published:** 2026-11-04

**Authors:** Frances Garraghan, Nicola Robinson, Jacqueline Sneddon, Ryan A Hamilton, Sarah Esberger, Andy Townsend, Susan A Mutua, Maria Dolores Niembro-Ortega, Winnie Owila, Matthew Song, Ashley Wilde, Khalid Yunis

**Affiliations:** British Society for Antimicrobial Chemotherapy, Birmingham, UK; British Society for Antimicrobial Chemotherapy, Birmingham, UK; Manchester University NHS Foundation Trust, Pharmacy Department, Wythenshawe Hospital Southmoor Road, Wythenshawe, Manchester, UK; British Society for Antimicrobial Chemotherapy, Birmingham, UK; British Society for Antimicrobial Chemotherapy, Birmingham, UK; British Society for Antimicrobial Chemotherapy, Birmingham, UK; Pfizer Inc. R&D I&I and Specialty Care Medical Affairs Department, Walton Oaks, Dorking Road, Tadworth, Surrey KT20 7NS, UK; Pharmacy Department, Gertrude's Children's Hospital, Gertrude's Children's Hospital, Nairobi, Kenya; Infectious Diseases Department, Instituto Nacional de Ciencias Médicas y Nutrición Salvador Zubirán, Instituto Nacional de Ciencias Médicas y Nutrición Salvador Zubirán, Mexico City, Mexico; Pharmacy Department, Gertrude's Children's Hospital, Gertrude's Children's Hospital, Nairobi, Kenya; Department of Pharmacy, Norton Healthcare, Louisville, KY, USA; Advocate Health, Enterprise Pharmacy Services, 3075 Highland Pkwy, Downers Grove, IL 60515, USA; American University of Beirut Medical Center, Division of Neonatology, Department of Pediatrics and Adolescent Medicine, National Collaborative Perinatal Neonatal Network NCPNN, American University of Beirut Medical Center, Beirut, Lebanon

## Abstract

**Background:**

Antimicrobial stewardship (AMS) is essential to addressing antimicrobial resistance, but its implementation remains variable, particularly in settings with resource limitations. Hospitals serving deprived or underserved populations often work with limited diagnostic capacity, workforce pressures and variable digital infrastructure, which shape stewardship delivery. The Antimicrobial Stewardship Disparities Programme (ASDP) was established to strengthen stewardship capability in such settings and to support progress towards accreditation under the Global AMS Accreditation Scheme (GAMSAS).

**Methods:**

A mixed-methods evaluation was undertaken across six centres in Botswana, Kenya, Lebanon, Mexico, Pakistan and the USA. Centres completed baseline and end of programme self-assessments against 17 AMS, infection prevention and control (IPC) and laboratory standards mapped to 4 pillars (structures, guidance, education and surveillance). Programme documentation was analysed to identify themes, contextual influences and barriers. All work was delivered within existing resources.

**Results:**

Centres achieved improvements across AMS, IPC and laboratory standards, with consistent progress in surveillance. Governance was strengthened through clearer AMS committee structures, and guidance improved through prescribing tools embedded in practice. Education and laboratory standards showed limited change, reflecting workforce pressures, lack of protected time and constrained diagnostic capacity. Three centres achieved GAMSAS accreditation. Qualitative findings highlighted strategies to advance stewardship within structural constraints, including surveillance systems, training and locally adapted guidance.

**Conclusions:**

Local aims, supported by a standards framework and mentorship, enabled centres to strengthen stewardship capability within existing resources. Workforce capacity, diagnostic access and digital infrastructure remain key determinants. Structured assessment and shared learning support scalable AMS in underserved settings.

## Introduction

Antimicrobial stewardship (AMS) is widely recognised as essential to addressing antimicrobial resistance (AMR),^1^ but its implementation remains variable, especially in settings with significant resource limitations.^2^ Hospitals that serve deprived populations often face limited diagnostic capacity, workforce shortages and variable digital infrastructure.^3^ These factors affect their ability to meet established stewardship standards and to deliver consistent practice across clinical, laboratory and infection prevention services.

Although there has been increasing global attention to stewardship frameworks and system-level improvement,^1^ there is limited comparative evidence on how stewardship programmes function in different health system contexts, particularly in settings affected by health inequality.^2^ International frameworks allow organizations to assess their stewardship activities against recognized standards, but cross-country applications of structured assessment tools remain uncommon.^4^

Health disparities are often most visible in low- and middle-income settings, where structural constraints and resource limitations can affect the delivery of stewardship activities.^3^ Similar patterns have been reported in evaluations of AMS programmes in resource-limited settings, with improvements in governance but persistent gaps in surveillance and guideline adherence.^5^ However, marked inequality also exists within high-income countries, where local populations may experience poorer access to services and worse health outcomes than neighbouring areas.^6^ This means that stewardship constraints can be shaped by local inequity rather than national income classification alone.

Populations affected by deprivation often experience a higher burden of infectious disease and worse outcomes from AMR, increasing the importance of effective AMS in these settings.^3,7^ Yet national or regional stewardship infrastructure is limited in many areas, leaving individual centres and clinicians to lead AMS activity with little coordinated support. In this context, networks that enable hospitals to share experience, tools and practical solutions can play an important role in strengthening stewardship practice.^8^

The Global AMS Accreditation Scheme (GAMSAS) developed by BSAC provides a structured framework that covers organizational stewardship, infection prevention and control (IPC) and laboratory capability and capacity. Many programmes in similar settings focus on laboratory, IPC or AMS improvements in isolation, making the integrated consideration of these domains within GAMSAS an important and novel approach.

Most published evaluations have been single-site studies or based in high-income settings,^4^ with limited use of qualitative analysis to understand the contextual factors that influence progress.^3^ This limits understanding of how stewardship programmes function in settings affected by health inequality and where resources are constrained.

This study formed part of the Antimicrobial Stewardship Disparities Programme (ASDP), which aimed to support improvement projects on the optimal use of antibiotics in hospitals with limited resources, in those serving underserved or deprived populations, or in both. The programme sought to strengthen stewardship capability via expert mentorship and to develop regional Centres of Excellence that could sustain and inspire further AMS development within countries. Following a structured application process, 10 centres (hospitals) were selected to participate, although four subsequently withdrew. The remaining centres received structured support to assess their stewardship capability and to design and deliver locally relevant improvement work. Many AMS strengthening initiatives rely on external funding for training or laboratory improvements, whereas the ASDP was intentionally delivered within existing resources, supporting reproducibility in settings with constrained capacity. Using accreditation as an incentive for stewardship improvement is uncommon in AMS strengthening initiatives, adding a further novel element to the programme design.

The evaluation used mixed methods to review progress against core stewardship standards and to analyse the activities, barriers and strategies described in routine programme documentation.

## Methods

### Design and setting

This study used a mixed-methods design drawing on self-assessment data and routinely collected programme documentation. It formed part of the ASDP, a collaboration between Pfizer and BSAC that supported six centres to review their stewardship activities and implement locally relevant improvement work. Centres were invited to apply to join the ASDP via an open grant call with all applications assessed by a multiprofessional team of experts in AMS from various regions of the world. The selected participating centres were based in Botswana, Kenya, Lebanon, Mexico, Pakistan and the USA. The USA centre was included because it supports a highly underserved population with structural constraints comparable to those seen in lower-income settings, aligning with the programme’s focus on health inequity rather than national income status. The centres implemented AMS across adult, paediatric and neonatal populations and represented regions with marked health inequality rather than a single income classification.

From February 2023 to January 2026, centres received support that included needs assessment, education, multidisciplinary team development, mentorship and evaluation. The tailored education provided to project teams focused on AMS learning aligned to each centre’s needs and was supported by generic training materials and expert mentorship. Centres took part in regular virtual mentorship meetings and biannual progress discussions, and teams were brought together annually to present their work and promote shared learning. The ASDP additionally offered organizations a pathway to accreditation under GAMSAS for those ready to undergo assessment against international standards.

### Assessment tool

All centres participating in the ASDP completed baseline and end of ASDP self-assessments of core AMS standards (as per the GAMSAS framework). The tool consisted of 17 standards covering organizational stewardship, IPC and laboratory capacity, mapped across four pillars: structures (St), education (Ed), guidance (Gu) and surveillance (Su). Centres reported whether each standard was fully met, partially met or not met at each time point. For reporting, the standards are presented as AMS1 to AMS10, IPC1 to IPC3 and Lab1 to Lab4. Centres are anonymized, and the detailed content of the standards is not reported due to the ASDP agreements.

### GAMSAS accreditation levels

GAMSAS awards accreditation at three levels based on the extent to which organizations meet the AMS, IPC and laboratory standards. Level 1, Level 2 and Level 3 reflect increasing alignment with the standards as assessed through the online surveys, supporting evidence and the site visit. In addition, organizations that demonstrate strong stewardship capability and provide mentorship or support to other hospitals may be designated as a Centre of Excellence, which is independent of the accreditation level.

### Data sources

Each centre defined its own aims at the outset of the ASDP and implemented interventions aligned to these aims. Progress was reviewed using biannual reports, quarterly meeting notes and materials from the virtual annual shared learning events. Qualitative data consisted of programme documentation generated by multidisciplinary teams across six centres. All data are presented in anonymized or aggregate form.

### Analysis

Self-assessment data were summarized by calculating the number of standards reported as fully met, partially met or not met at baseline and at the end of the ASDP for each centre. Changes in these categories were used to describe progress across the core standards. ASDP documentation was analysed manually using a structured extraction approach and grouped according to each centre’s aims to identify common themes, contextual influences and reported barriers. Qualitative findings supported interpretation of the self-assessment results.

This evaluation used routinely collected ASDP data and did not require ethical approval. All participating sites included in this report have given permission for use of their data in this report.

## Results

### Self-assessment of AMS, IPC and laboratory standards

The baseline and end of ASDP self-assessments showed variation in how individual centres met the AMS, IPC and laboratory standards. The heat map in Figure 1 summarizes, for each centre, the number of coded standards reported as fully met, partially met or not met at both time points.

**Figure 1.**
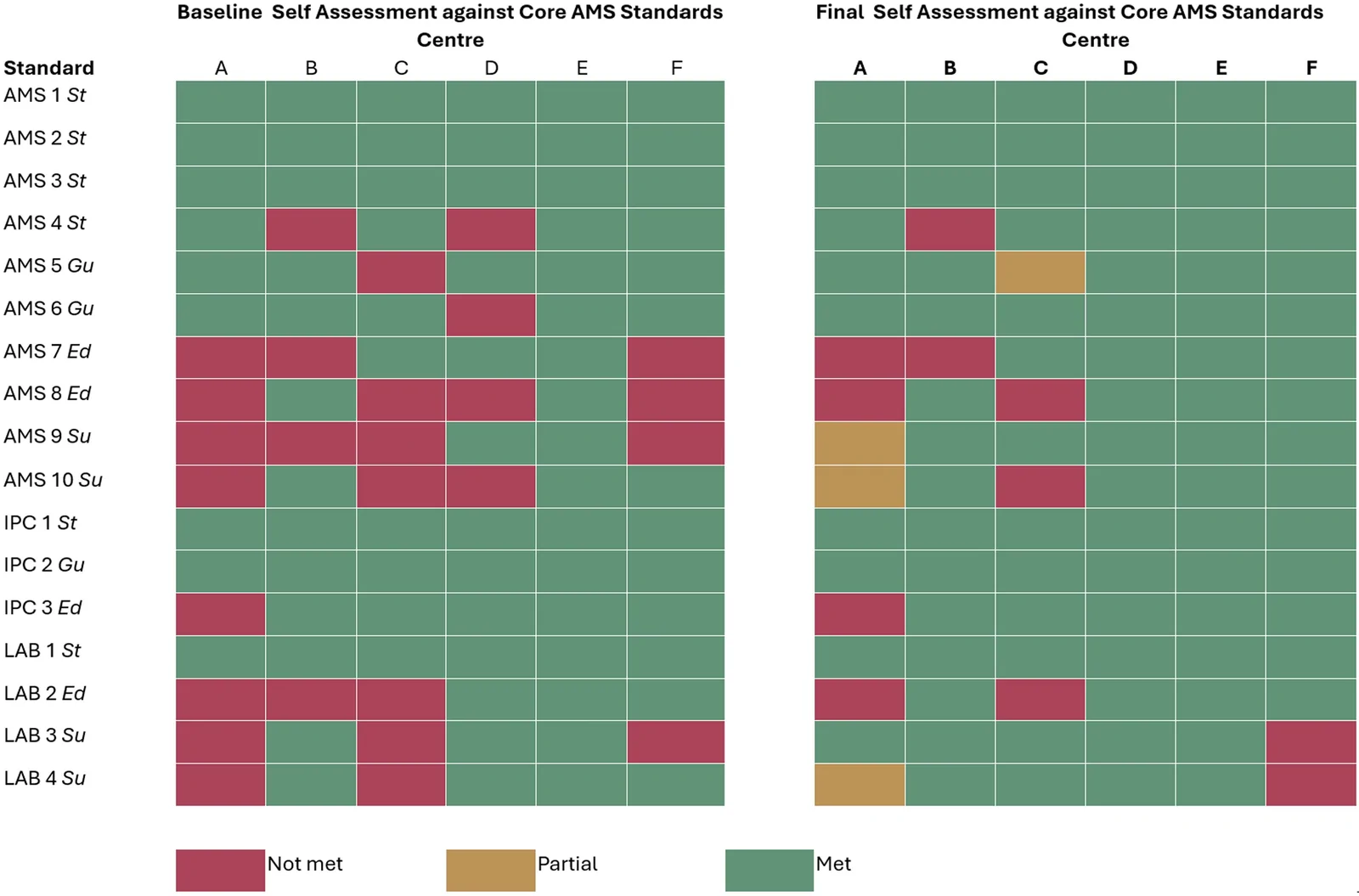
Comparison of baseline and end of ASDP status of AMS, IPC and laboratory standards across participating centres.

Across centres, several AMS standards showed improvement between baseline and end of ASDP assessments. Governance standards (AMS1 to AMS4, St) were relatively well established at baseline, with all centres achieving at least three of the four standards. Of the two centres not achieving AMS4 at baseline, one met the standard by the end of the ASDP. Guidance standards (AMS5 to AMS6, Gu) also improved, with both centres not meeting these at baseline reporting progress by the end of the ASDP. Education standards (AMS7 to AMS8, Ed) showed limited change, with three centres remaining below full achievement at the end of the ASDP. Surveillance standards (AMS9 to AMS10, Su) demonstrated the most consistent improvement, with all centres showing progress from baseline.

IPC standards which focused on structures (IPC1, St) and guidance (IPC2, Gu) were achieved by all centres at both time points. One centre did not achieve IPC3 (Ed) at either assessment.

Laboratory standards showed variation across centres. One centre demonstrated improvement in education (Lab2, Ed) and two centres demonstrated improvement in surveillance (Lab3 and Lab4, Su), supported by increased integration of laboratory data into routine monitoring systems.

One centre did not meet a laboratory surveillance indicator (Lab4 Su) at follow-up despite meeting it at baseline. This reflected the absence of updated surveillance data during the programme period rather than a reduction in capability, and participation in the ASDP highlighted the importance of strengthening laboratory data systems to support ongoing surveillance.

### Global AMS Accreditation Scheme

Three of the six centres achieved accreditation of their AMS programmes against the GAMSAS standards. The assessment was undertaken by AMS expert assessors and awarded by the independent accreditation panel of international AMS experts. See Table 1 for the outcome of the GAMSAS assessments.

**Table 1.** GAMSAS accreditation of ASDP centres

Centre	Accreditation level	Date
Instituto Nacional de Ciencias Médicas y Nutrición Salvador Zubirán—Mexico	Level 3	June 2024
Norton Healthcare Foundation—USA	Level 3 Centre of Excellence	July 2023
Gertrude’s Children’s Hospital—Kenya	Level 2	April 2026

### Qualitative findings

The qualitative data showed that ASDP centres strengthened stewardship capability through practical, locally adapted approaches. Despite operating in different health system contexts, common patterns were evident in how teams advanced AMS within structural and resource constraints.

#### Strengthening stewardship leadership

Centres formalized stewardship leadership by establishing or refining AMS committees, clarifying roles and integrating AMS oversight into existing governance structures. Multidisciplinary teams and focal persons supported coordination and continuity. These developments were achieved despite limited protected time and competing clinical demands, which affected the consistency of participation.

In one centre, inconsistent attendance at AMS committee meetings due to staffing pressures led to the introduction of a buddy system, where paired members shared responsibility for feedback and accountability.

In another, stewardship was embedded within an existing medication safety framework, supported by new clinical pharmacy roles that strengthened day-to-day AMS oversight.

#### Advancing surveillance through context-appropriate methods

All centres strengthened surveillance using approaches aligned to their digital and laboratory capacity. Some implemented electronic or cloud-based systems to support routine data submission and benchmarking, while others used manual or hybrid methods where electronic prescribing or automated reporting were not feasible. Several centres developed or updated antibiograms or introduced structured monitoring of antimicrobial use. These adaptations enabled surveillance progress even where digital infrastructure remained limited.

One centre developed a simple digital dashboard to improve antimicrobial stock transparency when a full electronic prescribing system was not available.

Another centre, specializing in neonatal-specific AMS, implemented a cloud-based surveillance system that enabled continuous submission of antimicrobial use data and routine benchmarking across multiple neonatal intensive care units.

#### Embedding clinical guidance to support consistent practice

Centres developed or adapted guidance to reduce variation in prescribing and support more consistent decision-making. This included disease-specific algorithms, treatment pathways, diagnostic initiation criteria and tools embedded into clinical workflows. These approaches helped address variation linked to diagnostic access, prescribing culture and limited specialist availability.

One centre introduced standardized initiation criteria for a high-risk clinical population, reducing unnecessary antibiotic exposure and improving consistency across units.

Another centre developed disease-based antibiotic algorithms and pre-treatment diagnostic guidelines to support more judicious prescribing in respiratory and critical care settings.

#### Building capability through targeted education

All centres delivered education activities across medical, pharmacy, nursing and laboratory teams. Approaches included structured training, targeted sessions, digital modules, multidisciplinary ward rounds and Train the Trainer models. Workforce pressures and high clinical demand affected attendance and continuity, but integrating training into existing meetings and using multidisciplinary teams supported sustained engagement.

In one centre, short-form educational materials and clinical cases were developed to support facilities with limited time for formal training, enabling consistent access to stewardship knowledge across a wide region.

In another centre, a mandatory AMS self-learning module ensured that all staff achieved a consistent baseline understanding of stewardship principles despite variable attendance at in-person sessions.

#### Enhancing diagnostic and laboratory support

Several centres strengthened laboratory capacity through the introduction of automated identification systems, development of antibiograms or establishment of surveillance for healthcare-associated infections. Where laboratory capacity remained limited, centres used simplified diagnostic criteria or focused on targeted clinical scenarios to support stewardship decisions.

One centre introduced automated identification and susceptibility testing, enabling the creation of an institutional antibiogram that informed prescribing decisions.

Two centres began contributing local AMR and microbiology data to national surveillance platforms, supporting participation in global reporting systems despite limited digital infrastructure.

#### Cross-cutting patterns

Across all sites, progress was shaped by disparities in workforce capacity, digital infrastructure, diagnostic capability and governance arrangements. Centres responded with practical, context-specific strategies including strengthened governance, tailored surveillance systems, embedded training and locally adapted guidance that enabled meaningful stewardship improvements within their constraints.

## Discussion

The ASDP demonstrated that centres can strengthen aspects of AMS, IPC and laboratory capability through locally selected project aims supported by a consistent standards framework. Variation in progress reflected differences in governance structures, workforce capacity and digital and diagnostic resources. Using baseline and follow-up assessments provided a practical way to understand each centre’s starting point and how their activities contributed to stewardship capability over time. Delivering all work within existing resources supported sustainability and highlighted the importance of strengthening the core systems that enable stewardship activity.

### ASDP model and the relationship between project aims and core standards

Centres joined the ASDP with different priorities and levels of AMS development. Each centre set its own project aim based on what was realistic within its staffing, governance and clinical pressures. The ASDP did not prescribe or rank these aims. They were chosen locally and reflected what each centre felt able to work on.

Centres completed a baseline self-assessment of the core AMS, IPC and laboratory standards at the start of the ASDP. These standards were not used to design the project aims, which were chosen locally, but they provided a clear picture of each centre’s starting point and a consistent way to assess how the work they carried out contributed to stewardship capability over time. The standards also offered a structured way to understand readiness for GAMSAS.

The ASDP did not provide funding. All work was carried out within each centre’s usual resources. This was intentional and aimed to support changes that could be sustained without relying on external financial support.

### Programme participation

Ten centres joined the ASDP. Six completed the full cycle and are included in this analysis. Four withdrew early due to local pressures, administrative changes or competing priorities. Their withdrawal was consistent with the structural pressures reported by the centres during the programme and provides important context for interpreting the findings.

### Progress against the core AMS, IPC and laboratory standards

The self-assessment data provided a structured way to understand how centres progressed across the AMS, IPC and laboratory domains. While there was overall improvement in compliance with the core AMS standards, the self-assessments showed variation in progress over the course of the programme. Governance standards were already relatively well established, and several centres strengthened committee structures and reporting lines during the ASDP. Guidance standards also improved, as centres were able to update or develop prescribing tools within existing processes.

Education standards showed limited change. Some centres reported that staffing pressures and lack of protected time made it difficult to deliver routine training. Surveillance standards improved more consistently. Centres introduced point prevalence surveys, prescribing audits and antimicrobial consumption monitoring, which were achievable without major infrastructure changes and could be integrated into existing clinical routines.

IPC structures and guidance were strong at baseline, which is consistent with the long-standing international focus on infection prevention and the regulatory requirements for IPC that exist in many health systems. Gaps in IPC and laboratory education mirrored the challenges seen in AMS education, where limited time and workforce capacity restricted the ability to deliver regular training. Variation in laboratory standards reflected differences in equipment, staffing and information systems, which shaped what was feasible within the timeframe of the ASDP.

### Insights from the project aims

The project aims covered a wide range of activities. Some centres focused on developing or updating prescribing guidance, others on establishing surveillance processes or delivering targeted education. The feasibility of these aims depended on existing governance arrangements, digital systems and workforce capacity. Centres with established AMS committees or clearer governance structures were often able to integrate project work into routine processes. Others faced constraints related to staffing, competing priorities or limited diagnostic and digital infrastructure.

The diversity of project aims showed how stewardship development is shaped by local context. Centres used practical approaches that fitted their operational realities. These activities provided insight into what could be achieved within existing systems and where structural barriers limited progress.

### How project work and the standards relate

Although the project aims were not defined by the GAMSAS standards, the standards helped show where project work contributed to stewardship capability. Improvements in governance, guidance and surveillance often aligned with the types of activities undertaken, even though this was not an explicit requirement. Areas with limited change, such as education and laboratory capacity, reflected broader structural constraints rather than lack of effort, which is consistent with findings from other global AMS initiatives.^9,10^

### Structural constraints, sustainability and disparities

Workforce limitations were a consistent barrier across AMS, IPC and laboratory domains. High clinical demand limited protected time and staff turnover restricted the ability to deliver training and sustain multidisciplinary engagement. Digital and diagnostic inequities also shaped what centres were able to implement. Centres with limited electronic prescribing or fragmented data systems relied on manual or hybrid approaches to surveillance. Despite these constraints, several centres demonstrated that meaningful surveillance could be achieved through simple tools or integration with national reporting systems.

The absence of external funding meant that all progress reflected what centres could achieve within their usual resources. This strengthened the sustainability of changes but also highlighted disparities in capacity. Centres with more established governance structures or stronger IPC and medication safety programmes were often better positioned to integrate stewardship activities into routine processes.

### Implications for scaling AMS in resource-limited or underserved settings

Future efforts to strengthen AMS in settings with health inequalities should recognize the value of locally selected project aims supported by a consistent standards framework. The structured standards framework provided a consistent reference point that could support national or regional stewardship programmes. Participation in surveillance systems such as WHO Global Antimicrobial Resistance and Surveillance System (GLASS) and stewardship structures aligned with existing governance arrangements can further support progress. Workforce development, protected time and sustainable training pathways remain essential for long-term progress.^1,2^ Strengthening digital and diagnostic infrastructure will also be important for reducing disparities in stewardship capability. These findings are consistent with experience from other global AMS initiatives, including the Fleming Fund^9^ and programmes led by Médecins Sans Frontières,^10^ which similarly highlight the importance of practical tools, shared learning and locally driven capability building in resource-limited settings.

### Limitations

This analysis is based on self-assessments completed by participating centres, which may be influenced by differences in interpretation or reporting. The ASDP supported locally selected project aims rather than a standardized intervention, meaning that activities and scope varied across centres. Four centres withdrew early, and their experiences are not included in the final analysis. The ASDP did not provide external funding, so progress reflects what was achievable within existing resources and may not capture the full range of potential activities in other settings.

The qualitative analysis was based on structured extraction of ASDP documentation and organized around the core standards and locally defined project aims. This approach identified common themes and contextual influences but did not capture every improvement described by centres. Some developments, including education and practice changes, did not alter the coded status of a standard and are therefore not fully reflected in the results.
